# N-cadherin promotes thyroid tumorigenesis through modulating major signaling pathways

**DOI:** 10.18632/oncotarget.14101

**Published:** 2016-12-22

**Authors:** Chenxing Da, Kexia Wu, Chenli Yue, Peisong Bai, Rong Wang, Guanjie Wang, Man Zhao, Yanyan Lv, Peng Hou

**Affiliations:** ^1^ Department of Endocrinology, The First Affiliated Hospital of Xi’an Jiaotong University, Xi’an 710061, P.R. China; ^2^ Department of Endocrinology, Shanxi Provincial Crops Hospital of Chinese People's Armed Police Force, Xi’an 710054, P.R. China; ^3^ Department of Endocrinology, Key Laboratory for Tumor Precision Medicine of Shaanxi Province, The First Affiliated Hospital of Xi’an Jiaotong University, Xi’an 710061, P.R. China

**Keywords:** thyroid cancer, N-cadherin, major signaling pathways, EMT process

## Abstract

Epithelial-mesenchymal transition (EMT), a crucial step in disease progression, plays a key role in tumor metastasis. N-cadherin, a well-known EMT marker, acts as a major oncogene in diverse cancers, whereas its functions in thyroid cancer remains largely unclear. This study was designed to explore the biological roles and related molecular mechanism of N-cadherin in thyroid tumorigenesis. Quantitative RT-PCR (qRT-PCR) and immunohistochemistry assays were used to evaluate N-cadherin expression. A series of *in vitro* studies such as cell proliferation, colony formation, cell cycle, apoptosis, migration and invasion assays were performed to determine the effect of N-cadherin on malignant behavior of thyroid cancer cells. Our results showed that N-cadherin was significantly upregulated in papillary thyroid cancers (PTCs) as compared with non-cancerous thyroid tissues. N-cadherin knockdown markedly inhibited cell proliferation, colony formation, cell migration and invasion, and induced cell cycle arrest and apoptosis. On the other hand, ectopic expression of N-cadherin promoted thyroid cancer cell growth and invasiveness. Mechanically, our data demonstrated that tumor-promoting role of N-cadherin in thyroid cancer was closely related to the activities of the MAPK/Erk, the phosphatidylinositol-3-kinase (PI3K)/Akt and p16/Rb signaling pathways in addition to affecting the EMT process. Altogether, our findings suggest that N-cadherin promotes thyroid tumorigenesis by modulating the activities of major signaling pathways and EMT process, and may represent a potential therapeutic target for this cancer.

## INTRODUCTION

Thyroid cancer is the most common endocrine malignancy and is one of the most rapidly increasing human cancers worldwide [1]. Thyroid cancer can be classified into differentiated thyroid cancer (DTC), poorly differentiated thyroid cancer (PDTC) and anaplastic thyroid cancer (ATC). DTC accounts for more than 90% of all thyroid cancers, including papillary thyroid cancer (PTC) and follicular thyroid cancer (FTC) [2]. Although the patients with DTC have good prognosis with the low mortality rate, there is a high recurrence rate and potential risk of differentiating into more aggressive and lethal thyroid cancer such as PDTC or ATC [3, 4]. Thus, we need make considerable efforts to better understand *molecular mechanisms underlying thyroid* tumorigenesis.

Tumor metastasis is the major cause of cancer-related deaths [5]. The development of cancer metastasis involves multiple steps. Epithelial-mesenchymal transition (EMT) is considered as an initial and necessary step by which epithelial cells lost their cell polarity and cell-cell adhesion, and gain migratory and invasive properties [6, 7]. The process of EMT can be regulated by a series of calcium-dependent, cell–cell adhesion molecules involved in epithelial maintenance, such as cadherin switching (that is, E-cadherin to N-cadherin) [8, 9]. A universal hallmark of EMT phenotype is the loss-of-function of E-cadherin and gain-of-function of N-cadherin in tumorigenesis [10, 11]. A growing body of evidence has shown that several transcription factors are aberrantly expressed in different types of cancer, contributing to the precess of EMT through negatively regulating E-cadherin transcription, including Snail, Twist and Slug [7, 11]. Moreover, N-cadherin has also been demonstrated to play a oncogenic role in bladder cancer [12], breast cancer [13], prostate cancer [14] and melanoma [15]. Moreover, interfering with its function may prove beneficial in multiple cancers [16–18]. However, the exact functions of N-cadherin in thyroid tumorigenesis remains largely unknown.

In this study, we found frequent overexpression of N-cadherin in primary PTCs as compared to control subjects. Functional studies demonstrated that N-cadherin downregulation significantly reduced *in vitro* oncogenic potential of thyroid cancer cells through modulating major signaling pathways and inhibiting the EMT process. On the other hand, ectopic expression of N-cadherin promoted the proliferative and invasive abilities of cancer cells, further supporting its tumor-promoting function in thyroid tumorigenesis.

## RESULTS

### Frequent overexpression of N-cadherin in thyroid cancer

**Figure 1:**
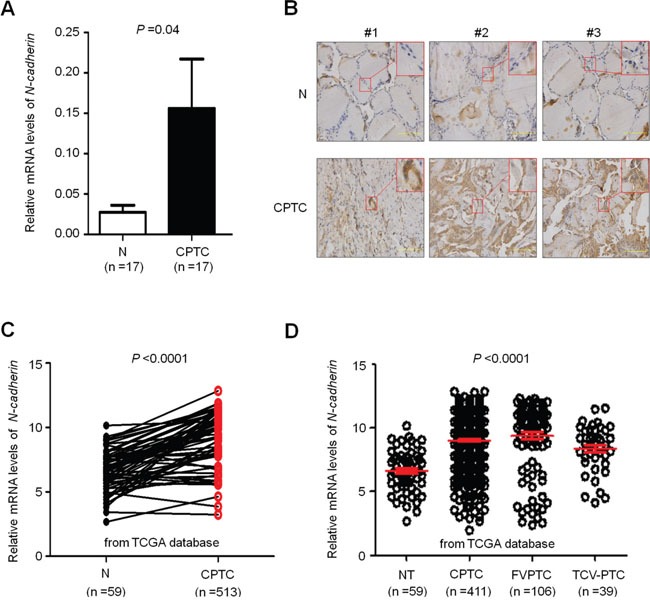
Increased expression of N-cadherin in PTCs A. qRT-PCR assay was performed to evaluate mRNA expression of N-cadherin in conventional PTCs (CPTCs) and their matched non-tumor thyroid tissues (N) (n =17). N-cadherin expression was normalized with 18S rRNA levels. B. Immunohistochemical (IHC) analysis was performed to evaluate N-cadherin expression. The positive staining was shown in a reddish-brown color. The sections were counterstained with Hematoxylin showing a blue color. The insert shows the magnified image of the area indicated by the red square. C and D. increased expression of N-cadherin in PTCs compared with normal thyroid tissues (NT) and matched normal thyroid tissues (N) in The Cancer Genome Atlas (TCGA) dataset. Horizontal lines indicate the median and interquartile range. CPTC, conventional PTC; FVPTC, follicular variant of PTC; TCV-PTC, tall-cell variant of PTC.

To explore the role of N-cadherin in thyroid cancer, we first evaluated its mRNA expression in 17 pairs of conventional papillary thyroid cancers (CPTCs) and matched non-cancerous thyroid tissues (control subjects) by quantitative RT-PCR (qRT-PCR). As shown in Figure 1A, N*-cadherin* was significantly increased in PTCs as compared to control subjects (*P* =0.04). Moreover, we further evaluated N-cadherin expression at protein levels by immunohistochemistry staining. As expected, we found that the expression of N-cadherin protein was markedly upregulated in CPTCs as compared to matched non-cancerous thyroid tissues (Figure 1B). These were further supported by The Cancer Genome Atlas (TCGA) dataset that mRNA expression of *N-cadherin* in PTCs was significantly upregulated as compared to matched normal thyroid tissues or normal controls regardless of pathological subtypes of PTC (Figure 1C and 1D).

### N-cadherin promotes thyroid cancer cell proliferation and colony formation

**Figure 2:**
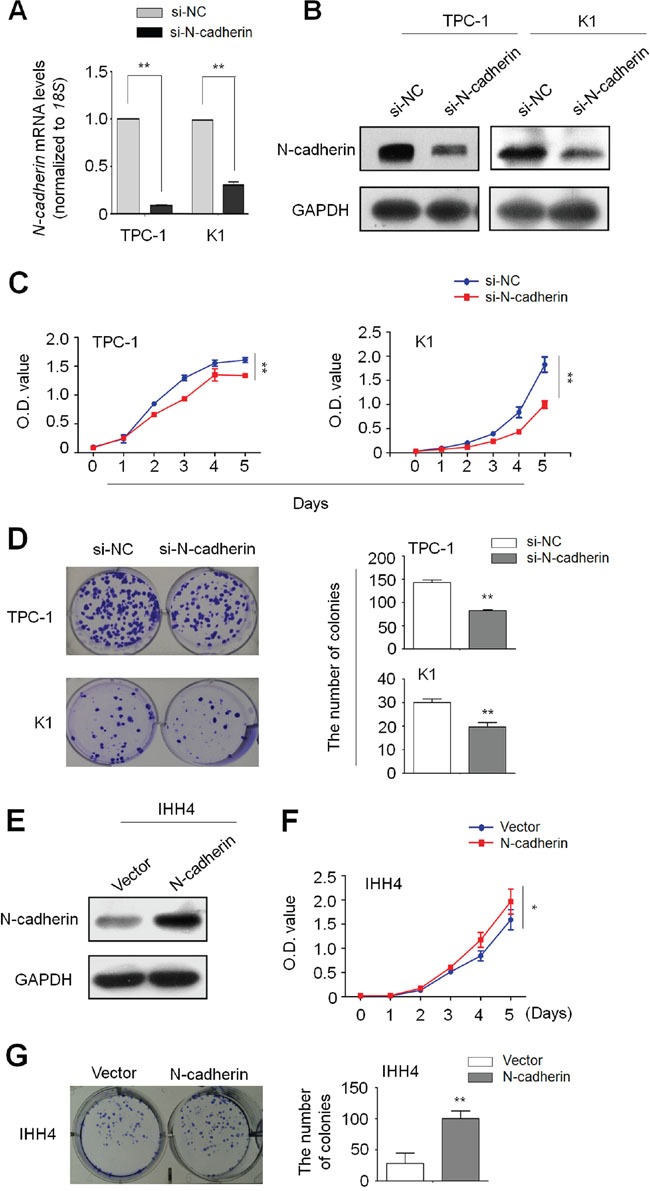
Increased cell proliferation and colony formation in thyroid cancer cells by N-cadherin Knockdown of N-cadherin mRNA A. and protein B. by siRNA targeting N-cadherin (si-N-cadherin) in TPC-1 and K1 cells was validated by qRT-PCR and western blot assays, respectively. 18S rRNA was used as a normalized control for qRT-PCR assay. GAPDH was used as loading control for western blot assay. The data were presented as mean ± SD. C. N-cadherin knockdown inhibited thyroid cancer cell proliferation. The data were presented as mean ± SD. D. N-cadherin knockdown inhibited colony formation of thyroid cancer cells. Left panel shows the representative images of colony formation in cells transfected with the indicated siRNAs. Quantitative analysis of colony numbers is shown in right panel. The data were presented as mean ± SD. E. Ectopic expression of N-cadherin in IHH4 cells was validated by western blot analysis. N-cadherin overexpression significantly enhanced cell proliferation F. and colony formation G. in IHH4 cells. The data were presented as mean ± SD. Statistically significant differences were indicated: *, P <0.05; **, P<0.01.

Given that N-cadherin is a well-known EMT marker and frequently overexpressed in PTCs, we speculate that it may play an oncogenic role in thyroid tumorigenesis. We first analyzed mRNA levels of N-cadherin in eight thyroid cancer cell lines by conventional RT-PCR. As shown in Supplementary Figure 1, TPC-1 and K1 cells showed high levels of N-cadherin, whereas IHH4 cells showed very low levels of N-cadherin. Thus, we tested the growth-inhibitory effect by silencing N-cadherin expression in TPC-1 and K1 cells using specific siRNA. qRT-PCR (Figure 2A) and western blot (Figure 2B) assays were performed to validate N-cadherin knockdown by specific siRNA sequences. Our data showed that N-cadherin knockdown significantly inhibited thyroid cancer cell proliferation and colony formation as compared to the control (Figure 2C and 2D). On the other hand, ectopic expression of N-cadherin in IHH4 cells (Figure 2E) dramatically enhanced cell proliferation and colony formation ability as compared with empty vector-transfected cells (Figure 2F and 2G). Taken together, these findings suggest the growth-promoting role of N-cadherin in thyroid cancer cells.

### N-cadherin promotes cell cycle progression and inhibits cell apoptosis in thyroid cancer cells

**Figure 3:**
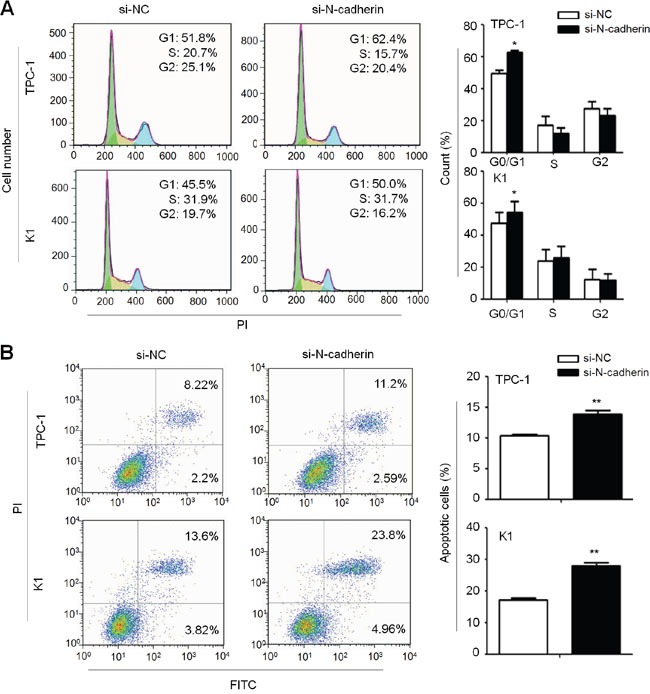
Induction of thyroid cancer cell cycle arrest and apoptosis by N-cadheirn knockdown A. TPC-1 and K1 cells were transiently transfected with the indicated siRNAs. DNA content was measured by flow cytometry to determine cell cycle fractions. The fraction of cells in each cell cycle phase was indicated in the figures. B. Early apoptotic cells (Annexin-V-FITC positive and PI negative) in bottom right quarter and late apoptotic cells (Annexin-V-FITC and PI positive) in top right quarter were measured by cytometry analysis using Annexin V-FITC Detection Kit, respectively. The data were presented as mean ± SD of values from three independent experiments. Statistically significant differences were indicated: *, P<0.05; **, P<0.01.

Next, we tested the impact of altered expression of N-cadherin on contribution of cell cycle and apoptosis in thyroid cancer cells. As shown in Figure 3A, cell cycle was arrested at the G0/G1 phase in si-N-cadherin transfected cells as compared with si-NC transfected cells. The percentage of G0/G1 phase was increased from 49.55% ± 1.9% to 62.65% ± 1.3% in TPC-1 cells (*P* =0.019) and from 47.35 ± 6.9% to 54.32 ± 0.9% in K1 cells (*P* =0.036), respectively. Moreover, N-cadherin knockdown significantly induced cell apoptosis as compared to the control (Figure 3B). The percentage of apoptotic cells was increased from 10.34 ± 0.2% to 13.84 ± 0.6% in TPC-1 cells (*P* =0.005), and from 17.21 ± 0.9% to 27.89 ± 1.7% in K1 cells (*P* =0.002), respectively. On the other hand, ectopic expression of N-cadherin in IHH4 cells caused a decrease in the percentage of G0/G1 phase as compared to the control (61.53 ± 0.64% vs. 65.13 ± 1.52%, *P* =0.03) (Supplementary Figure 2A), and restoring N-cadherin expression in IHH4 cells also inhibited cell apoptosis as compared to the control (2.67 ± 0.60% vs.5.60 ± 0.93%, *P* =0.01) (Supplementary Figure 2B).

### N-cadherin promotes thyroid cancer cell migration and invasion

**Figure 4:**
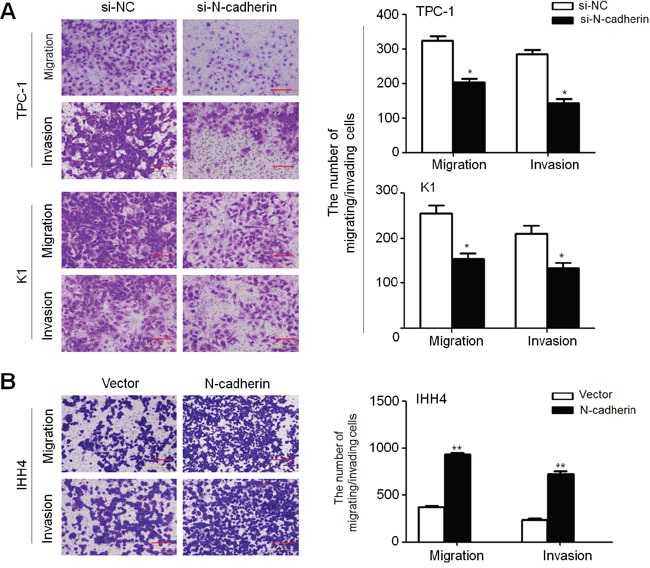
Increased cell migration and invasion in thyroid cancer cells by N-cadherin A. N-cadherin knockdown inhibited cell migration and invasion in TPC-1 and K1 cells. B. Ectopic expression of N-cadherin in IHH4 cells enhanced cell migration and invasion. The representative images of migrated/invaded cells (left panels). Histograms, corresponding to upper panels, show mean ± SD of cell numbers from three independent assays (right panels). Statistically significant differences were indicated: *, P<0.05; **, P<0.01.

Given that N-cadherin has been widely reported to be involved in cancer metastasis, we attempted to evaluate the effect of N-cadherin on migration and invasion potential of thyroid cancer cells. Our data showed that cell migration and invasion potential was significantly decreased in si-N-cadherin transfected TPC-1 and K1 cells as compared to control cells (Figure 4A). On the other hand, N-cadherin overexpression in IHH4 cells significantly enhanced the ability of cell migration and invasion (Figure 4B). Altogether, our findings suggest that there is a close link between N-cadherin overexpression and metastatic potential in thyroid cancer.

### N-cadherin contributes to tumorigenesis by modulating the activities of major signaling pathways in thyroid cancer cells

**Figure 5:**
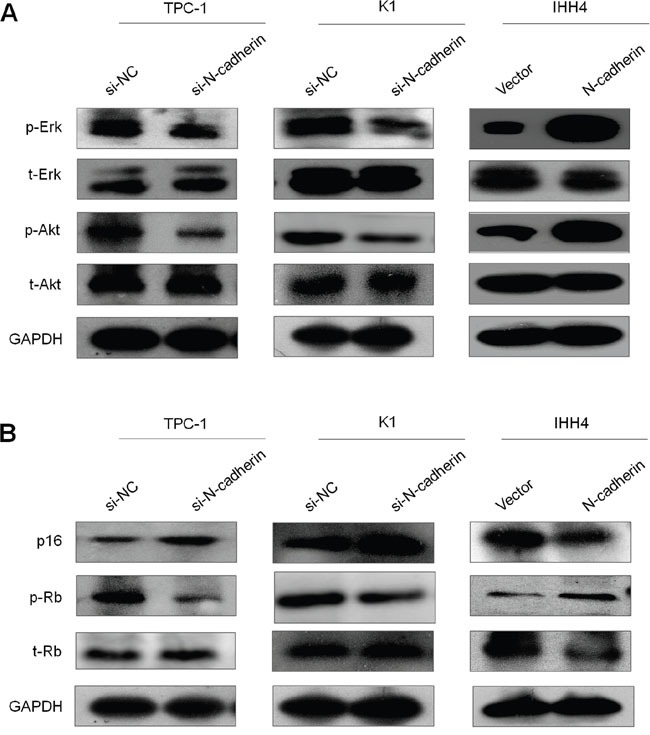
N-cadherin modulates the activities of the MAPK/Erk, PI3K/Akt and p16/Rb signaling pathways in thyroid cancer The lysates from the indicated cells were subjected to western blot analysis. A. The antibodies against phospho-Akt (p-Akt), total Akt (t-Akt), phospho-Erk (p-Erk) and total Erk (t-Erk) were used to determine the effect of N-cadherin kncokdown or overexpression on the activities of the PI3K/Akt and MAPK/Erk pathways. B. The antibodies against p16, phospho-Rb (p-Akt), total Rb (t-Rb) were used to determine the effect of N-cadherin kncokdown or overexpression on the activity of the p16/Rb pathway. GAPDH was used as a loading control.

To explore the mechanisms underlying tumor-promoting activity of N-cadherin in thyroid cancer, we tested the effect of altered expression of N-cadherin on the activities of MAPK/Erk and PI3K/Akt signaling pathways, which play key roles in thyroid cancer occurrence and progression [19]. Out results showed that N-cadherin knockdown significantly reduced phosphorylation of Erk (p-Erk) and Akt (p-Akt) in TPC-1 and K1 cells, whereas N-cadherin overexpression substantially increased phosphorylation of Erk and Akt in IHH4 cells (Figure 5A). These findings suggest that N-cadherin promotes thyroid tumorigenesis through activating the MAPK/Erk and PI3K/Akt pathways.

It is clear that the p16/Rb signaling pathway controlling cell cycle progression is commonly disrupted in malignancies including thyroid cancer [20]. Thus, we also tested the impact of N-cadherin on the activity of this pathway. Our results showed that N-cadherin knockdown in TPC-1 and K1 cells dramatically elevated the expression of p16 and decreased the phosphorylation of Rb (p-Rb). On the other hand, N-cadherin overexpression in IHH4 cells decreased the expression of p16 and increased the levels of p-Rb (Figrue 5B). These data indicate that N-cadherin contributes to thyroid tumorigenesis through promoting cell cycle progression via p16/Rb signaling.

### N-cadherin promotes cell metastasis through regulating the process of epithelial-mesenchymal transition (EMT) and the expression of metastasis-related genes in thyroid cancer

**Figure 6:**
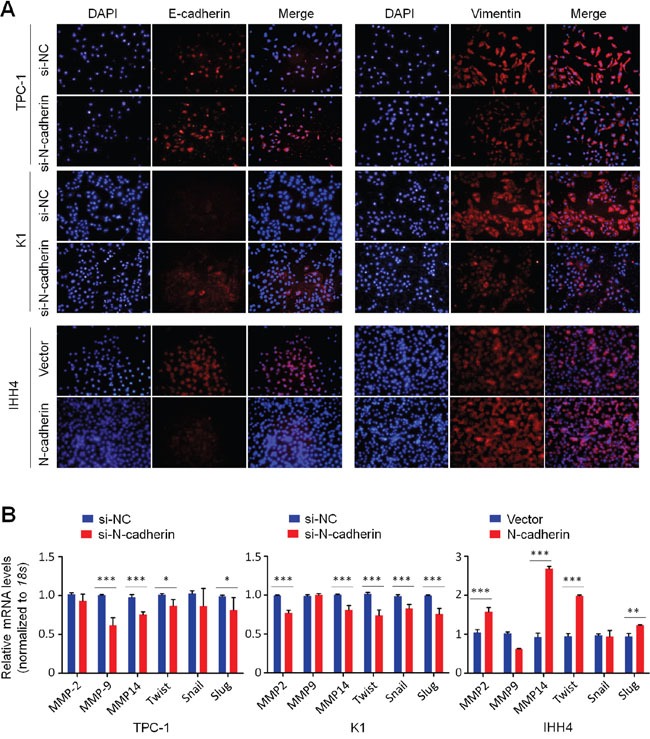
Effect of N-cadherin on the process of EMT and the expression of metastasis-associated genes in thyroid cancer cells A. Immunofluorescence staining was performed to assess the effect of N-cadherin on the expression of EMT-associated markers E-cadherin and Vimentin in thyroid cancer cells. Red color represents target protein fluorescence and blue color represents Hoechst33342 staining for nuclei. B. qRT-PCR assay was performed to test the effect of N-cadherin on the expression of metastasis-associated genes in thyroid cancer cells. Expression levels of these genes were normalized with 18S rRNA levels. Data were presented as mean ± SD. *, P <0.05; **, P<0.01; ***, P <0.001.

Given that N-cadherin is a key marker for EMT and involved in cancer metastasis, we next demonstrated the effect of N-cadherin on EMT process through regulating the expression of other EMT-relevant proteins such as E-cadherin and Vimentin. As expected, immunofluorescence assay demonstrated that N-cadherin depletion increased the expression of epithelial marker E-cadherin and decreased the expression of mesenchymal marker Vimentin in TPC-1 and K1 cells (Figure 6A). Conversely, N-cadherin overexpression in IHH4 cells significantly inhibited E-cadherin expression and increased Vimentin expression (Figure 6A). It is well-known that Twist, Snail and Slug are transcription factors that negatively regulates E-cadherin expression, contributing to the EMT process in tumorigenesis [21]. Thus, we attempted to investigate the effect of N-cadherin on their expression in thyroid cancer cells. The results indicated that N-cadherin knockdown significantly inhibited the expression of *Twist*, *Snail* and *Slug* in at least one cell line, whereas ectopic expression of N-cadherin in IHH4 cells dramatically upregulated the expression of *Twist* and *Slug* (Figure 6B). Given a strong link between matrix metalloproteinases (MMPs) and tumor metastasis [22], we also tested the effect of altered expression of N-cadherin on the expression of *MMP*-*2*, -*9* and -*14* in thyroid cancer cells. Also shown in Figure 6B, N-cadherin knockdown caused a dramatic decrease in the transcription of these three genes in at least one cell line. On the other hand, ectopic expression of N-cadherin in IHH4 cells significantly increased *MMP-2* and *MMP-14* expression. Altogether, our data suggests that N-cadherin promotes thyroid cancer cell metastasis by regulating the EMT process and the transcription of metastasis-associated genes.

## DISCUSSION

It is the fact that EMT plays a critical role in tumor metastasis, triggered by diverse stimuli that can result in a stable epithelial to mesenchymal transition in cancer cells [23]. In this study, we demonstrated that N-cadherin, a key marker of EMT, was frequently overexpressed in primary PTCs as compared to control subjects. In addition to enhancing cell metastasis via inducing the EMT process, we also showed that N-cadherin promoted thyroid tumorigenesis through promoting cell proliferation, colony formation and cell cycle progression.

To better understand tumor-promoting function of N-cadherin in thyroid cancer, we first tested the effect of N-cadherin on aberrant signaling of the MAPK/Erk and PI3K/Akt pathways. These two pathways have been widely investigated in malignancies and demonstrated to play critical roles in tumorigenesis. Thus, they become important therapeutic targets for malignancies including thyroid cancer [3, 24]. Our data showed that N-cadherin knockdown remarkably blocked the activities of these two pathways in thyroid cancer cells, whereas N-cadherin overexpression enhanced their activities. This is supported by the previous studies that N-cadherin promotes tumorigenesis *in vitro* and *in vivo* through activating these two pathways [25, 26]. Mechanically, tumor-promoting activity of N-cadherin results probably from the interaction with certain growth factor receptor via their extracellular domains. For example, N-cadherin physically interacts with fibroblast growth factor receptor (FGFR) to stabilize FGFR-1 and activate Erk1/2 cascade, and subsequently increase MMP-9 production [27, 28]. Taken together, N-cadherin contributes to thyroid tumorigenesis through activating the MAPK/Erk and PI3K/Akt signaling pathways via interacting with certain growth factor receptors.

The p16/Rb axis has been well demonstrated to critically regulate G1 to S phase progression, and is frequently disrupted in malignancies including thyroid cancer. In this study, we demonstrated that N-cadherin knockdown significantly upregulated the levels of p16, and subsequently decreased the levels of Rb phosphorylation through specifically inhibiting Cdk4 and Cdk6 kinase activity. On the other hand, N-cadherin overexpression in thyroid cancer cells decreased the levels of p16 and increased the levels of Rb phosphorylation, promoting cell cycle progression. There is also evidence showing that the MAPK/Erk signaling can regulate p16 expression through epigenetic mechanism such as promoter methylation in cancer cells [29]. Collectively, these observations suggest that N-cadherin promotes thyroid tumorigenesis through modulating the activity of p16/Rb signaling pathway via Erk-dependent or -independent ways.

It is clear that cadherin switching (such as transition from E-cadherin to N-cadherin) regulates the cellular migratory and invasion ability during the EMT process. In line with this, our data showed a significant increase of E-cadherin and reduction of Vimentin staining in the cell membrane of N-cadherin knockdown cells. On the other hand, ectopic expression of N-cadherin decreased E-cadherin expression and increased Vimentin expression in IHH4 cells. A number of studies have demonstrated that EMT regulatory factors Twist, Snail and Slug can be modulated by major signaling pathways including MAPK/Erk and PI3K/Akt pathways [30–32]. Indeed, our results demonstrated that N-cadherin knockdown remarkably inhibited the expression of these genes in thyroid cancer cells, whereas N-cadherin overexpression increased their expression. These data indicate that N-cadherin promotes the EMT process through transcriptionally regulating the EMT-associated markers such as E-cadherin, Vimentin and itself via the activation of MAPK/Erk and PI3K/Akt pathways. Newly synthesized N-cadherin is subsequently translocated to cellular membrane where it interacts with some growth factor receptors to activate their downstream signaling pathways, contributing to tumorigenesis including thyroid cancer. Additionally, considering the importance of MMPs in tumor metastasis, we also investigated the impact of N-cadherin on the transcription of MMP-2,-9, and -14 in thyroid cancer cells. The results demonstrated that N-cadherin knockdown significantly inhibited their expression, whereas ectopic expression of N-cadherin increased their expression. These findings suggest that N-cadherin promotes cell metastasis at least in part through regulating the expression of some MMPs genes.

**Figure 7:**
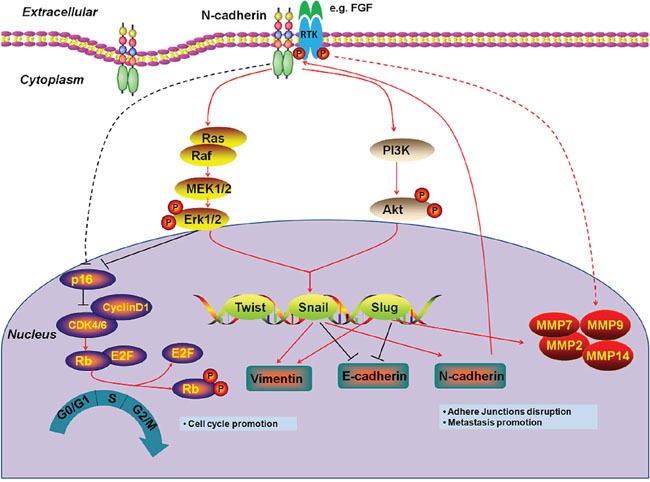
Schematic model of molecular mechanisms underlying tumor-promoting effect of N-cadherin in thyroid cancer Increased expression of N-cadherin stabilizes certain growth factor receptors (e.g., FGFR) to activate their downstream signaling pathways including MAPK/Erk and PI3K/Akt pathways through interacting with their extracellular domains in thyroid cancer cells. The activation of MAPK/Erk and PI3K/Akt cascades upregulates the EMT regulatory factors such as Twist, Snail and Slug. These transcription factors then promotes the process of EMT through transcriptionally regulating the EMT-associated markers such as E-cadherin, Vimentin and N-cadherin and MMPs production. Newly synthesized N-cadherin is subsequently translocated to cellular membrane where it plays a tumor-promoting function through interacting with some growth factor receptors. In addition, N-cadherinalso promotes cell cycle progression through modulating p16/Rb pathway via Erk-dependent or –independent mechanisms.

In summary, we observed that N-cadherin was frequently overexpressed in primary PTCs. Our data are consistent with a model (Figure 7) in which N-cadherin promotes thyroid tumorigenesis through enhancing cell proliferation, colony formation, metastasis, and cell cycle arrest progression, and inhibiting cell apoptosis via activating major signaling pathways and inhibiting EMT process in thyroid cancer.

## MATERIALS AND METHODS

### Clinical samples

With the approval of institutional review board and human ethics committee, a total of 17 primary PTCs and their matched non-cancerous thyroid tissues were randomly obtained from the First Affiliated Hospital of Xi’an Jiaotong University. None of these patients received any therapeutic intervention before surgery. All of the tissues were histologically examined by two senior pathologists at Department of Pathology of the Hospital based on World Health Organization (WHO) criteria.

### RNA extraction and quantitative RT-PCR (qRT-PCR)

Total RNA from the tissues and cell lines were isolated by TRIzol reagent following manufacturer's instruction (Takara Inc., Dalian, China), and the cDNA was prepared using Prime Script RT reagent Kit (Takara Inc., Dalian, China). Quantitative RT-PCR (qRT-PCR) was carried out on a CFX96 Thermal Cycler Dice™ real-time PCR system (Bio-Rad Laboratories, Inc., CA) using SYBR Premix Ex Taq™ (Takara Inc., Dalian, China). The mRNA expression of the indicated genes was normalized to *18S* rRNA cDNA. Each sample was run in triplicate. The primer sequences were presented in Supplementary Table 1.

### Immunohistochemistry (IHC)

IHC analysis was performed to evaluate N-cadherin expression in PTCs. In brief, the paraffin-embedded sections were deparaffinized and rehydrated in a graded series of ethanol, and washed in distilled water. After antigen retrieval and blocking, the sections were incubated with anti-N-cadherin antibody (Abcam) overnight at 4°C. After washing, the slides were then incubated with biotinylated goat anti-rabbit IgG (ZSGB- BIO, Beijing, P.R. China). Immunodetection was performed with the Streptavidin-Peroxidase system (ZSGB-bio, Beijing, P.R. China) according the manufacture's protocol.

### Cell culture, short interfering RNA (siRNA) and expression plasmid transfection

Human thyroid cancer cell lines TPC-1, K1 and IHH4 were provided by Dr. Haixia Guan (The First Affiliated Hospital of China Medical University, Shenyang, China). TPC-1, K1, IHH4 cells were cultured at 37°C in RPMI 1640 or DMEM medium with 10% fetal bovine serum (FBS) (Invitrogen Technologies, Inc, CA), respectively. Oligonucleotides of siRNA targeting N-cadherin(si-N-cadherin) and control siRNA(si-NC) were obtained from Gene Pharma (Shanghai, China) and the sequences were presented in Supplementary Table 2. Cells were transfected at 70% confluence using Lipofectamine 2000 (Invitrogen, Grand Island, NY), with a final siRNA concentration of 50nM. Specific oligonucleotides with maximal knockdown efficiency were selected among three different sequences until use. All silencing experiments were performed in three replicates. To construct N-cadherin expression plasmid, the full-length open reading frame (ORF) of human *N-cadherin* gene without stop codon TGA was amplified and then cloned into pcDNA3.1(-) with a Myc-His tag. Cells were transfected with different plasmids using X-tremeGENE HP DNA Transfection Reagent (Roche) according to the instructions of the manufacturer.

### Western blot analysis

The indicated cells were lysed in RIPA buffer containing protease inhibitors. Supernatants were collected and subjected to 10% SDS-PAGE, and transferred onto PVDF membranes (Roche Diagnostics, Mannheim, Germany). The membranes were then incubated overnight with primary antibodies. Anti-N-cadherin was purchased from Abcam Biotechnology, Inc. Anti-phospho-Erk1/2, anti-phospho-Akt^Ser473^, anti-total Erk and anti-total Akt were purchased from Bioworld Technology, co, Ltd. Anti-p16, anti-phospho-Rb and anti-Rb antibodies were purchased from Abcam. Anti-GAPDH was purchased from Abgent, Inc. This was followed by incubation with species-specific HRP-conjugated secondary antibodies from ZSGB-BIO, and antigen-antibody complexes were visualized using the Western Bright ECL detection system (Advansta, CA).

### Cell proliferation and colony formation assay

Cell proliferation was measured by the MTT assay. Briefly, the indicated cells were seeded and cultured in 96-well plates. At the indicated times, 20 μl of 0.5 mg/ml MTT (Sigma, Saint Louis, MO) was added into the medium and the plates were further incubated for 4 h, followed by adding 150 μl of DMSO. The plates were then read on a micro plate reader using a test wavelength of 570 nm and a reference wavelength of 670 nm. All MTT assays were done in triplicate.

Colony formation assay was performed using monolayer culture. The indicated cells were cultured in 6-well plates. The medium was refreshed every 3 days. After 14 days of culture, surviving colonies (≥50 cells per colony) were fixed with methanol and stained with 0.5% crystal violet, and the colonies were then counted. Each experiment was performed in triplicate.

### Cell cycle and apoptosis assays

For cell cycle analysis, the indicated cells were synchronized by serum starvation for 12 h and induced to reenter the cell cycle by an exchange of 10% FBS. Cells were then harvested at 48h after replacing the medium and fixed in ice-cold 70% ethanol at 4°C overnight. Subsequently, cells were stained with propidium iodide solution (50 μg/mL propidium iodide, 50 μg/mL RNase A, 0.1% Triton-X, 0.1 mM EDTA), and subjected to FACS analysis (BD Biosciences, NJ).

For apoptosis analysis, the indicated cells were harvested, washed with PBS, suspended in binding buffer, and sequentially stained with Annexin V-FITC Detection Kit (Roche Applied Science, Penzberg, Germany) by flow cytometry. Each experiment was performed in triplicate.

### Cell migration and invasion assays

Cell migration and invasion assays were evaluated by transwell chambers (8.0 μm pore size; Millipore, MA). To assess invasive ability of cancer cells, the chambers were pre-coated with Matrigel (BD Bioscience, NJ). The indicated cells were starved overnight and then cultured in the upper chamber. Medium with 10% FBS (1 ml) was added to the lower chamber. After 24h incubation, non-migrating/non-invading cells in the upper chamber were removed using a cotton swab, and migrating/invading cells were then fixed in 100% methanol and stained with crystal violet solution (0.5% crystal violet in 2% ethanol). The number of migrating/invading cells was expressed as the average number of cells per microscopic field over five fields.

### Immunofluorescence staining (IF)

The process of immunofluorescence staining was similarly performed as described previously [33]. In brief, the indicated cells were grew on coverslips in 6-well plates. Cells were then fixed with 4.0% formaldehyde, and blocked with 5% goat serum. Next, the coverslips were incubated at 4°C with primary antibodies overnight. Anti-E-cadherin and anti-Vimentin antibodies were purchased from Epitomics, Inc. Subsequently, the coverslips were incubated with Cy3-conjugated goat anti-rabbit secondary antibody (Bioss, Beijing, P.R. China) and dried, dyed with Hoechst33342, and fixed in glycerol. The images were obtained with an Olympus IX71 microscope (Olympus, Tokyo, Japan), and color mergence was performed using Image J image software (ImageJ version 1.44p, NIH, MD).

### Statistical analysis

*N-cadherin* expression in cancer tissues and control subjects were compared by a standard 2-tailed *t* test and Mann–Whitney *U* test using SPSS statistical package (16.0, Chicago, IL). The data are expressed as mean ± standard deviation (SD) of the mean as indicated. *P*< 0.05 was considered statistically significant.

## SUPPLEMENTARY MATERIALS FIGURES AND TABLES


