# Supplementary material for: N-cadherin promotes thyroid tumorigenesis through modulating major signaling pathways
Source: Oncotarget. 2016 Dec 22;8(5):8131–42. doi: 10.18632/oncotarget.14101 (PMC5352388; doi:10.18632/oncotarget.14101)
Supplement: Supplementary file 1 [file oncotarget-08-8131-s001.pdf]

N-cadherin promotes thyroid tumorigenesis through modulating major signaling pathways

SUPPLEMENTARY FIGURES AND TABLES

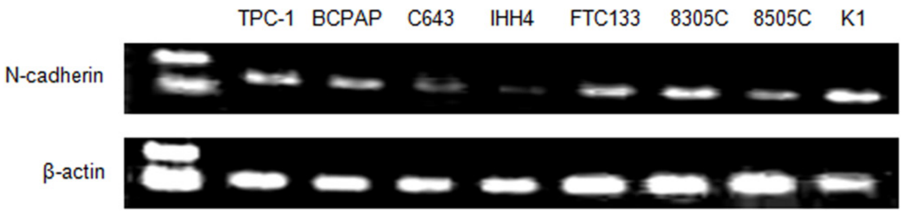

Supplementary Figure 1: N-cadherin mRNA expression in eight thyroid cancer cell lines. N-cadherin mRNA expression in thyroid cancer cell lines was determined by conventional RT-PCR assay. β-actin was used as a load control.

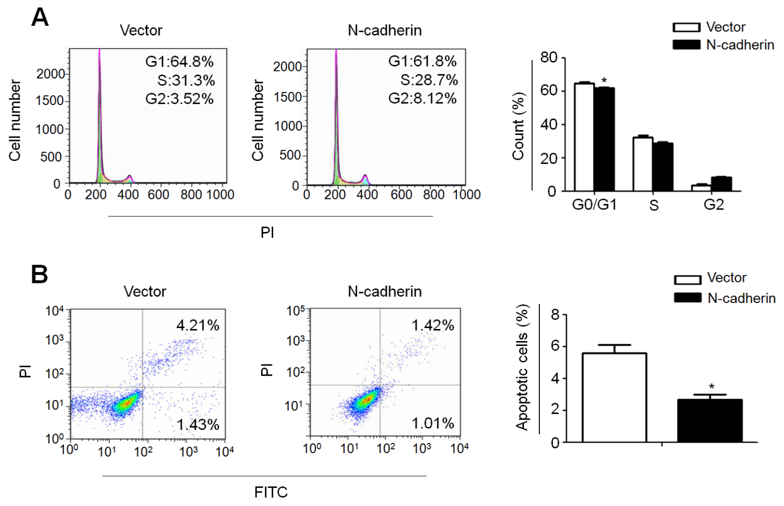

Supplementary Figure 2: N-cadheirn promotes cell cycle progression and inhibits cell apoptosis in IHH4 cells. A. IHH4 cells were transiently transfected with the indicated constructs. DNA content was measured by flow cytometry to determine cell cycle fractions. The fraction of cells in each cell cycle phase was indicated in the figures. **B.** Early and late apoptotic cells were measured by cytometry analysis using Annexin V-FITC Detection Kit. The data were presented as mean ± SD of values from three independent experiments. Statistically significant differences were indicated: *, P <0.05.

Supplementary Table 1: qRT-PCR primers used in this study

Genebank (ID)	Genes	Forward primer (5'-3')	Reverse primer (5'-3')	Product length (bp)
NM_001792.4	*N-cadherin*	GTGCATGAAGGACAGCCTCT	CCACCTTAAAATCTGCAGGC	100
NM_000474.3	*Twist*	TCCATTTTCTCCTTCTCTGGAA	CCTTCTCGGTCTGGAGGAT	127
NM_005985.3	*Snail*	CCTTCTCTAGGCCCTGGCT	AGGTTGGAGCGGTCAGC	106
NM_003068.4	*Slug*	TCGGACCCACACATTACCTT	GCAGTGAGGGCAAGAAAAAG	121
NM_004530.5	*MMP-2*	GGAAAGCCAGGATCCATTTT	ATGCCGCCTTTAACTGGAG	103
NM_004994.2	*MMP-9*	ACGACGTCTTCCAGTACCGA	GCACTGCAGGATGTCATAGG	128
NM_004995.3	*MMP-14*	GTGGTCTCGGACCATGTCTC	GGTAGCCATATTGCTGTAGCC	146
R_003286.2	*18S*	CGCCGCTAGAGGTGAAATTC	CTTTCGCTCTGGTCCGTCTT	52

Supplementary Table 2: The sequences of siRNAs used in this study

si-RNAs	Sequences (5'-3')
si-N-cadherin (sense)	GACCAUCACUCGGCUUAAUTT
si-N-cadherin (antisense)	AUUAAGCCGAGUGAUGGUCTT
si-NC (sense)	UUCUCCGAACGUGUCACGUTT
si-NC (antisense)	ACGUGACACGUUCGGAGAATT
